# Detection of glypican-1 (GPC-1) expression in urine cell sediments in prostate cancer

**DOI:** 10.1371/journal.pone.0196017

**Published:** 2018-04-19

**Authors:** Douglas H. Campbell, Maria E. Lund, Aline L. Nocon, Paul J. Cozzi, Mark Frydenberg, Paul De Souza, Belinda Schiller, Jennifer L. Beebe-Dimmer, Julie J. Ruterbusch, Bradley J. Walsh

**Affiliations:** 1 Minomic International Ltd, Sydney, New South Wales, Australia; 2 St George and Sutherland Clinical School, the University of New South Wales, Sydney, New South Wales, Australia; 3 Department of Surgery, St George Hospital, Sydney, New South Wales, Australia; 4 Epworth Healthcare, Melbourne, Victoria, Australia; 5 Department of Surgery, Monash University, Melbourne, Victoria, Australia; 6 Department of Medical Oncology, Liverpool Hospital, Sydney, New South Wales, Australia; 7 School of Medicine, University of Western Sydney, Sydney, New South Wales, Australia; 8 Karmanos Cancer Institute, Detroit, Michigan, United States of America; 9 Department of Oncology, Wayne State University School of Medicine, Detroit, Michigan, United States of America; Duke University School of Medicine, UNITED STATES

## Abstract

While measurement of serum prostate specific antigen (PSA) is an important screening tool for prostate cancer, new biomarkers are necessary for better discrimination between presence and absence of disease. The MIL-38 monoclonal antibody is specific for the membrane glycoprotein glypican 1 (GPC-1) and binds to prostate cancer tissue. Urine is known to be a source of cellular material. Thus, we hypothesized that detection of GPC-1 in urine cellular material may identify individuals with prostate cancer. Urine samples from patients with prostate cancer, benign prostatic hyperplasia (BPH), or normal controls were collected and cell sediments prepared. GPC-1-positive cells were detected using a MIL-38 immunofluorescence assay (IFA) and samples were classed positive or negative for GPC-1 expressing cells. Assay sensitivity and specificity, stratified by PSA, was reported. A total of 125 patient samples were analyzed (N = 41 prostate cancer; N = 37 BPH; N = 47 normal controls). The use of MIL-38 to detect GPC-1 by IFA discriminated between prostate cancer and BPH urine specimens with a sensitivity and specificity of 71% and 76%, respectively. Assay specificity increased with increasing PSA, with the highest specificity (89%) for patients with PSA ≥4 ng/ml. At lower PSA (<2 ng/ml) specificity decreased, as evidenced by a greater number of false positives in this concentration range. The odds ratio (OR) and 95% confidence intervals (CIs) for GPC-1-positive cells in patients with prostate cancer, adjusted for PSA, was greatest at the lowest serum PSA (<2 ng/ml; OR = 13.4; 95% CI: 4.0–44.7) compared with no adjustment for PSA (OR = 6.4; 95% CI: 2.8–14.9). The use of MIL-38 for detection of GPC-1 may be a useful tool for detection of prostate cancer.

## Introduction

Current clinical guidelines for the diagnosis of prostate cancer recommended by the American Urological Association comprise an initial screening to measure serum prostate-specific antigen (PSA) and a digital rectal examination (DRE) to detect any abnormalities in the prostate. The patient is offered the option of undergoing a biopsy for a definitive diagnosis in the case of abnormal findings [1]. The use of PSA as a screening tool has been shown to be beneficial for the early detection of aggressive tumors, decreasing the mortality associated with prostate cancer [2]. However, despite the high sensitivity of PSA measurement, the specificity of this biomarker is poor, with a positive predictive value of only 32% for men with PSA values >4 ng/ml [3]. Consequently, a relatively high PSA warrants further investigation requiring invasive biopsies that are often negative for prostate cancer [4]. As a result, there is a clinical need for other diagnostic tools that are non-invasive and can better discriminate between the presence and absence of prostate cancer. A diagnostic assay with higher specificity could be used as an adjunct to PSA.

Urine cytology is a well-established, non-invasive technique to detect abnormal cells shed from the prostate, bladder, and urinary tract [5, 6]. However, cytological assessment relies heavily on the pathologist’s experience and skill, which may affect the reproducibility of the findings [6, 7]. The specificity of urine cytology is very high, but sensitivity can be quite low, particularly for conditions of the prostate [5]. The analysis of urine cell sediments (UCS) for expression of various biomarkers has received considerable attention as a potential diagnostic approach. Recently, expression analysis of different prostate cancer biomarkers, such as TMPRSS2:ERG (fusion of the androgen receptor-responsive promoter of the serine protease gene, TMPRSS2, with the transcription factor ERG), alpha-methylacyl-CoA racemase (AMACR), and prostate cancer gene 3 (PCA3), in urine sediments has been reported [8–11]. Indeed, the detection of biomarkers in the urine may support cytological assessment for diagnosis.

The MIL-38 antibody (previously named BLCA-38) targets the glypican-1 (GPC-1) antigen [12]. GPC-1 is a heparan sulfate proteoglycan that is found attached to the cell surface via a glycophosphatidylinositol (GPI) anchor. The MIL-38 antibody targets GPC-1 on several prostate cancer cell lines and, importantly, on prostate cancer tissue [12, 13]. Thus, detection of GPC-1 on prostate cancer cells using MIL-38 may be a useful tool for the diagnosis of prostate cancer. We have developed an immunofluorescence assay (IFA) incorporating MIL-38 that can detect GPC-1 expressing cells in sediments from urine samples. We report here the use of MIL-38 recognition of GPC-1 in UCS to distinguish between patients with prostate cancer and those with no cancer (both normal controls and patients with benign prostatic hyperplasia or hypertrophy (BPH). In addition, patient PSA values were correlated with the presence of GPC-1 expressing cells in the urine to evaluate the combined diagnostic power of the two tests. These data suggest the potential use of GPC-1 as a biomarker for prostate cancer.

## Materials and methods

### Patient population

Urine samples were collected from patients at 3 Australian hospitals between 2010 and 2011 upon approval of the study by the University of New South Wales Ethics Committee (HREC 10174). All patients provided written informed consent for collection of samples and provision of their clinical data.

A total of 120 patients were planned for enrollment in the study, and 203 patient samples were collected. This comprised approximately equal numbers of healthy volunteers or men post-prostatectomy (74), patients with biopsy-confirmed BPH (69), and patients with biopsy-confirmed localized prostate cancer (60). Patients included in the study were matched for age. All PSA values were provided by the patient’s clinician.

### Sample collection and preparation

Patients with prostate cancer had catheterized urine collected (38 patients) or provided voided urine (3 patients)–no notable difference in cell abundance was observed between catheterized and voided urine. Control and BPH patients provided voided urine. All voided samples were collected midstream and approximately 50–100 ml was collected from each patient.

Urine samples were neutralized with 50% v/v 200mM phosphate buffer (pH 7.0). Cells were collected by centrifugation (500xg), and washed 3 times with phosphate-buffered saline (Dulbecco’s PBS). Cell pellets were resuspended in 50μl of PBS and 1μl was inspected using inverted microscopy to evaluate cell density. Samples were processed for IFA if >1 cell was visible during this inspection; if no cells were observed, the sample was discarded. Approximately equal numbers of samples were discarded from each cohort (prostate cancer, normal controls and BPH). Cell samples were prepared in triplicate and spotted onto epoxy-coated slides (8 mm wells), air-dried and fixed with ice-cold acetone for 3 minutes at -20°C. After fixation, slides were processed immediately or stored at 4°C and then processed within one week.

The prostate cancer cell line DU-145 (American Type Culture Collection; ATCC; HTB-81) was used as a positive control and the GPC-1 low expressing bladder cancer cell line C3 was included as a negative control (Prof. P Russell, Queensland University of Technology, Queensland, Australia) [13]. Cultured tumor cell lines were detached from culture flasks by incubation with 2mM EDTA in PBS for 10 to 15 min and centrifuged at 180xg for 5 min. Cells were washed, enumerated, and slides prepared, as described above.

For analysis of GPC-1 expression in UCS by western blot, healthy control and prostate cancer samples (protocol approved by Macquarie University Human Ethics Committee; reference number 52015500707) were prepared as described above for the IFA assay. Cell pellets were then lysed in Membrane Extraction Buffer (20mM HEPES, 0.5mM EDTA, 0.5% Triton X100, pH 7.5, protease inhibitor cocktail; a buffer known to extract membrane GPC-1) and incubated with rotation for 30 min at RT. Samples were then centrifuged (16000 x g, 15 min, 4°C), and the supernatant frozen (-20°C; urine cell extract). Urine cell extracts were thawed and immunoprecipitated using MIL-38 conjugated Dynabeads together with Heparinase I (Sigma H2519; calcium chloride 5mM was added) by incubation overnight at 4°C. GPC-1 was eluted from the beads by boiling in XT Sample Buffer (BioRad, 1610791) diluted to 2x in H_2_O. For DU-145 control cell extracts, DU-145 cells were lysed in Buffer I (20mM MES, 0.5mM EDTA, 0.005% Triton X100, pH 6.5) and incubated rotating for 10 min at RT. The samples were then centrifuged (16 000 x g, 15 min, 4°C) and the supernatant discarded. The pellet was then lysed in Membrane Extraction Buffer, and samples were treated as described above for UCS, from this point onward, except that Heparinase I treatment was performed after immunoprecipitation.

Urine cell sediment membrane extracts were loaded on 4–12% Novex Bolt Bis-Tris Plus gels (BioRad) such that equivalent total cell numbers were loaded for each sample. Protein was transferred to a nitrocellulose membrane using a Trans-blot Turbo (BioRad). The blot was blocked with blocking buffer (5% skim milk w/v diluted in PBS-tween20 0.1% v/v; PBS-T) for 40 min at RT, followed by incubation with biotinylated MIL-38 (0.5μg/ml diluted in blocking buffer) O/N at 4°C. Following 3 washes in PBS-T, binding of MIL-38 was detected by incubation with Streptavidin-HRP (Thermofisher Scientific; 1/15,000, diluted in blocking buffer) for 1h at RT. The blot was washed x 3 with PBS-T, once with PBS, and then developed with Clarity western ECL substrate (BioRad) and imaged using the ImageQuant LASmini4000 (GE Life Sciences). The UCS lanes were exposed for 5 min, while the DU-145 and marker lanes were exposed for 2 min.

### Immunofluorescence assay

All steps were performed at room temperature in a humidified chamber. Dry samples were rehydrated by soaking the slides in PBS for 3 minutes, followed by blocking with 5% skim milk in PBS for 30 minutes. For direct IFA, MIL-38 antibody (Minomic International Ltd, Sydney, Australia) was labelled with fluorescein isothiocyanate (FITC) (Celllabs Pty Ltd, Sydney, Australia) according to manufacturer’s instructions. Samples were incubated with MIL-38-FITC (10μg/ml) in blocking solution for 1 h. Cells were washed 3 times with PBS, counterstained with 2-(4-amidinophenyl)-1H-indole-6-carboxamidine (DAPI) and mounted with Fluoroshield™ (Sigma #F6182). Due to the autofluorescence in some samples, an indirect IFA was used to increase the signal intensity. For indirect IFA, cells were incubated with MIL-38 (10 μg/ml) in blocking solution for 1 h, washed with PBS then stained with goat anti-mouse IgG (H+L) (Life Technologies, Carlsbad, CA, USA) conjugated to FITC or Alexa Fluor™ 488 (10μg/ml in blocking solution) for 30 minutes protected from light. Cells were washed 3 times with PBS, counterstained with DAPI and mounted with Fluoroshield™. Each patient sample was prepared in triplicate. One slide was used stained with secondary antibody alone, and the other two slides were stained with MIL-38. All slides were inspected by manual scanning of the whole slide under epifluorescence microscopy for the presence of MIL-38-stained cells.

Samples were classified positive if >1 cell was positive for MIL-38 staining in both slides. The sample was discarded if the secondary antibody control slide was positive or if the slides had <10 cells per slide.

### Statistical analysis

For each study arm the mean and standard deviation for age at study participation was calculated and compared using analysis of variance (ANOVA) and pair-wise t-tests. The distribution between study arms for PSA (categorized as <2 ng/ml, 2 to <4 ng/ml, and ≥4 ng/ml) and GPC-1 IFA type (direct or indirect) were compared using chi-square tests. Test performance statistics (sensitivity and specificity) were calculated for prostate cancer patients compared with both normal and BPH patients collectively, and BPH patients only. Test statistics were also calculated by stratifying the PSA levels for patients with prostate cancer or BPH using 2 different clinically relevant PSA cutoff points: 2 ng/ml and 4 ng/ml. To estimate the combined effect of PSA and MIL-38, logistic regression was used to estimate both the odds ratio (OR) and the 95% confidence intervals (95% CIs) for detecting cancer compared with both the normal control patients and BPH groups. The ORs were presented for the association between MIL-38 and cancer, along with the association adjusted for PSA (dichotomous variable based on either 2 ng/ml or 4 ng/ml cutoff points). All statistical analyses were completed using SAS software, version 9.4 (Cary, NC, USA).

## Results

### Patient demographics

A total of 125 patients were included in the study for analysis. The mean±SD (standard deviation) age of patients in the BPH group (68.5±8.6 years) was significantly greater compared with patients in the prostate cancer (64.3±6.7 years; *P* = 0.026) or normal patient groups (63.7±9.6 years; *P* = 0.045; Table 1). As expected, PSA values were significantly higher in the prostate cancer and BPH groups (*P*<0.001, respectively) compared with the normal groups and significantly more elevated in the prostate cancer group compared with the BPH group (*P*<0.001; see Table 1).

**Table 1:** Patient demographics and clinical characteristics.

	Prostate cancer (N = 41)	BPH (N = 37)	Normal (N = 47)	P-value Between Patient Groups			
				Prostate Cancer vs BPH vs Normal	Prostate Cancer vs BPH	Prostate Cancer vs Normal	BPH vs Normal
**Age**							
N samples	40	34	29				
				0.041	0.026	0.774	0.045
Mean (SD)	64.3 (6.7)	68.5 (8.6)	63.7 (9.6)				
**PSA, N (%)**^a^							
<2ng/ml	5 (12)	14 (38)	39 (98)				
2 to <4ng/ml	8 (20)	14 (38)	1 (3)	<0.001	<0.001	<0.001	<0.001
≥4ng/ml	28 (68)	9 (24)	0				
**GPC-1 IFA Assay Type, N (%)**^b^							
Direct	35 (90)	8 (22)	4 (9)				
				<0.001	<0.001	<0.001	0.096
Indirect	4 (10)	29 (78)	42 (91)				
**Urine Source, N (%)**^c^							
Catheterised	35 (92)	0	0				
Void	3 (8)	37 (100)	46 (100)				
**Gleason score (Biopsy), N (%)**							
3+3	13 (32)						
3+4	19 (46)						
4+4	1 (2)						
4+5	6 (15)						
Unknown	2 (5)						
**Gleason score (Surgery**^d^**), N (%)**							
3+3	7 (17)						
3+4	16 (39)						
4+3	5 (12)						
7 (NOS)	2 (5)						
4+4	2 (5)						
4+5	4 (10)						
Unknown	5 (12)						

BPH, benign prostatic hyperplasia; GPC-1, Glypican-1; IFA, immunofluorescence assay; NOS, not otherwise specified; PSA, prostate-specific antigen; SD, standard deviation.

^**a**^PSA unknown for 7 patients.

^b^Assay type unknown for 3 samples.

^c^Urine source unknown for 4 samples.

^d^Radical prostatectomy.

### Evaluation of the MIL-38 IFA assay for the detection of GPC-1 expressing cells in urine cell sediments

**Fig 1:**
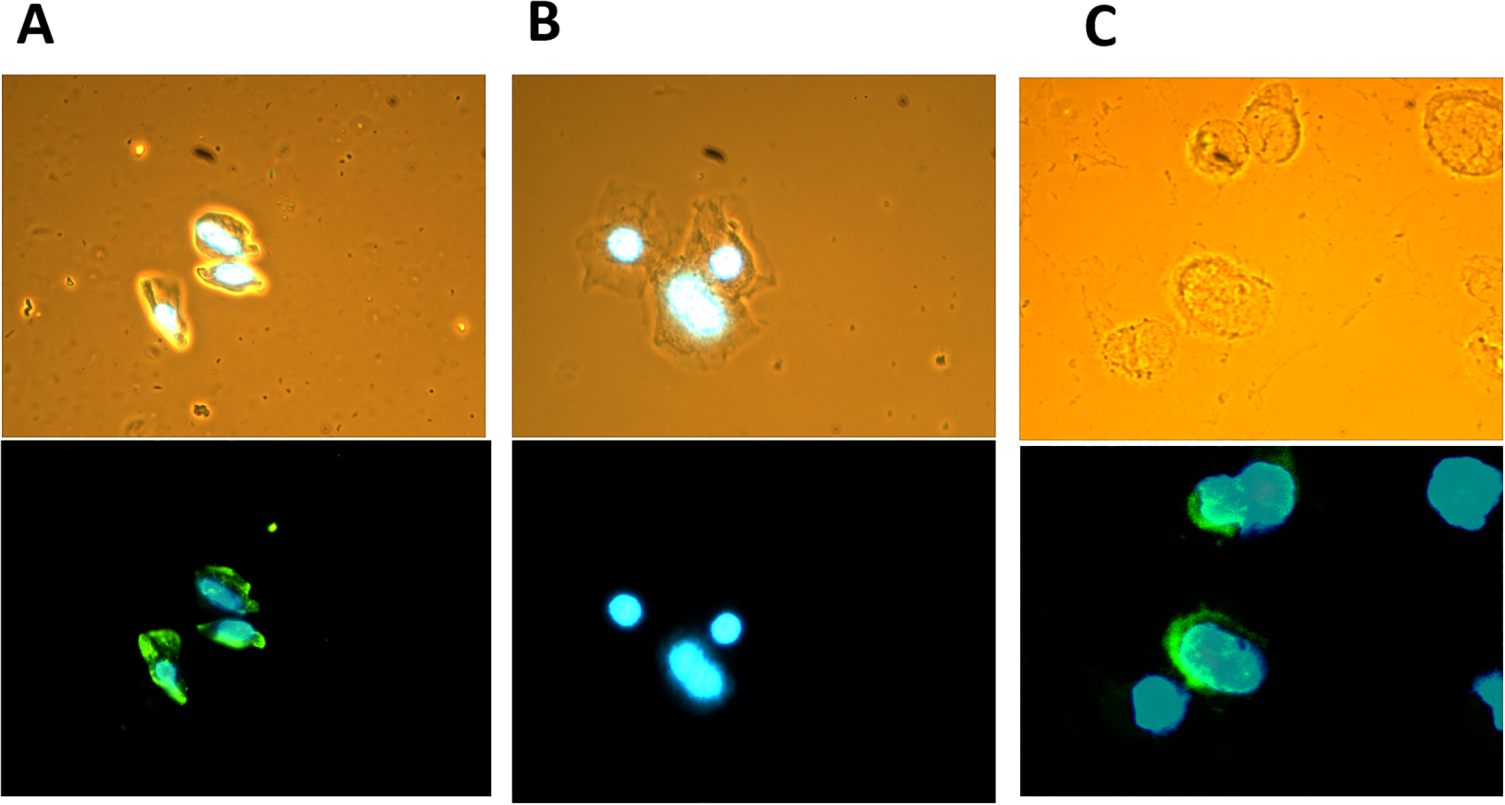
Typical microscopic findings for a positive sample in MIL-38 immunofluorescence assay of cell sediments from patient urine. **Top**: bright field and DAPI-merged image; **bottom:** goat anti-mouse IgG (H+L)-Alexa Fluor™ 488 and DAPI fluorescence-merged image. **A.** Positive cells in a prostate cancer patient sample. **B.** Negative cells in the same prostate cancer patient sample. **C.** DU-145 prostate cancer cell line.

To demonstrate the use of the MIL-38 IFA assay to detect cell surface GPC-1, control slides of the GPC-1 high cell line DU-145 were stained with the assay. GPC-1 expression was detected in the DU-145 control slides (Fig 1).

**Fig 2:**
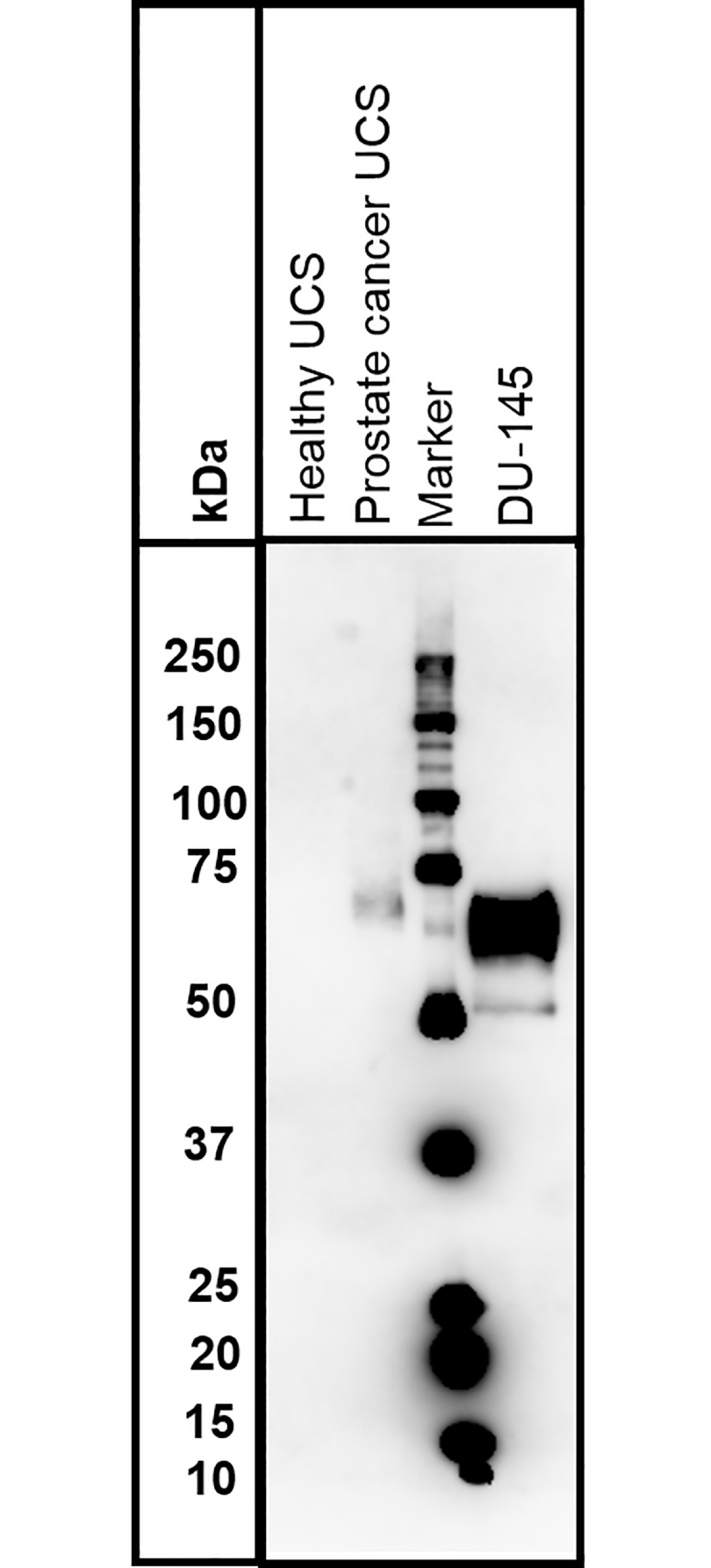
Detection of glypican-1 in the membrane fraction of prostate cancer urine cell sediment by western blot. The blot shows immunoprecipitates from membrane fractions of urine cell sediments (UCS) prepared from a healthy control and patient with prostate cancer, or GPC-1^+^ DU-145 cells. MIL-38 was used to probe the western blot.

The IFA assay was then evaluated for its ability to detect GPC-1 expression in UCS and expression of GPC-1 was detectable in patient samples (Fig 1). Moreover, in a given GPC-1 positive sample, some cells showed strong MIL-38 staining of GPC-1, while other cells were negative for MIL-38, suggesting specificity of MIL-38 for cells expressing the antigen (Fig 1). To confirm detection of GPC-1 in UCS, western blot analysis was performed using MIL-38 (that recognizes GPC-1) as detection antibody. Membrane enriched fraction of UCS immunoprecipitated with MIL-38 from prostate cancer was compared to healthy control UCS. After normalization for cell number, no GPC-1 was detected for healthy control UCS, but a band representing cell surface expressed GPC-1 (~70kDa; equivalent to that seen for GPC-1 positive DU-145 cell extracts; a slight difference in size between UCS and DU-145 extract attributed to different heparinase treatment protocols is observed) was detected for prostate cancer (Fig 2).

The use of an IFA assay requires detection of fluorescence above background that may be caused by cellular autofluorescence and/or non-specific binding of primary or secondary antibodies. Thus, patient cell sediment preparations were screened for cellular autofluorescence and fluorescence in no primary antibody control samples, and excluded from analysis if background fluorescence was present. The cellular density of the UCS sample was critical to the use of the IFA assay. Cell preparations that did not reach the cut off described in Materials and Methods were excluded from analysis. As a result of exclusion based on background fluorescence and cell density, only 125/203 (61.6%) patient samples were analysed. This included 41 samples from patients with prostate cancer, 37 from patients with BPH, and 47 from control patients.

### Specificity and sensitivity of MIL-38 staining for prostate cancer

MIL-38-positive staining cells were detected in the majority of prostate cancer samples (29/41; 71%: (Table 2), but were present only in a minority of BPH (9/37; 24%) or control (14/47; 30%) samples. In addition, MIL-38-positive cells were detected in samples for all Gleason grades.

**Table 2:** Sensitivity and specificity of GPC-1 detection for prostate cancer in cell sediments from patient urine samples using MIL-38: Comparison of prostate cancer samples with BPH and normal samples.

Patient Status	Prostate Cancer vs Normal + BPH		Sensitivity	Specificity
Prostate Cancer (N = 41)	True Positive	False Negative	71%	73%
	29	12		
Normal + BPH (N = 84)	False Positive	True Negative		
	23	61		
	Prostate Cancer vs BPH			
Prostate Cancer (N = 41)	True Positive	False Negative	71%	76%
	29	12		
BPH (N = 37)	False Positive	True Negative		
	9	28		

BPH, benign prostatic hyperplasia; GPC-1, glypican 1; MIL-38, anti-GPC-1 monoclonal antibody; N, number of samples.

Detection of GPC-1 discriminated between the prostate cancer cohort and the combined cohorts of BPH and normal control specimens with a sensitivity and specificity of 71% and 73%, respectively (Table 2). Similarly, the IFA assay discriminated between prostate cancer and BPH groups with a sensitivity and specificity of 71% and 76%, respectively. When stratified by serum PSA values, the assay specificity (89%) was highest in patients with higher PSA values (i.e. ≥4 ng/ml). However, specificity decreased at lower PSA (i.e., <2 ng/ml), as evidenced by a greater number of false positives in this concentration range (Table 3). The odds of a positive finding using the MIL-38 IFA in a patient with prostate cancer, adjusted for PSA, was greatest at the lowest serum PSA values (<2 ng/ml) with OR = 13.4 (95% CI: 4.0, 44.7) compared with 6.4 (95% CI: 2.8, 14.9) without adjustment for PSA (Table 4).

**Table 3:** Sensitivity and specificity of MIL-38 IFA stratified by PSA value in cell sediments from urine samples for patients with and without prostate cancer.

PSA Value	MIL-38 IFA result	Cancer Present?		Sensitivity	Specificity
		Yes (N = 41)	No (N = 77)^a^		
<2ng/ml	Positive	5	17		
				100%	68%
	Negative	0	36		
≥2ng/ml	Positive	24	4		
				67%	83%
	Negative	12	20		
<4ng/ml	Positive	10	20		
				77%	71%
	Negative	3	48		
≥4ng/ml	Positive	19	1		
				68%	89%
	Negative	9	8		

IFA, immunofluorescence assay; MIL-38, anti-GPC-1 monoclonal antibody; N, number of patients; PSA, prostate-specific antigen.

^a^PSA value unknown for 7 patients.

**Table 4:** Logistic regression odds ratios for a positive MIL-38 IFA result adjusted for patient PSA value.

	Odds Ratio	95% CI
GPC-1 IFA	6.4	2.8–14.9
GPC-1 IFA (adjusted for PSA value <4 ng/ml)	10.2	3.2–32.8
GPC-1 IFA (adjusted for PSA value <2 ng/ml)	13.4	4.0–44.7

CI, confidence interval; GPC-1, glypican 1; IFA, immunofluorescence assay; MIL-38, anti-GPC-1 monoclonal antibody; PSA, prostate-specific antigen.

## Discussion

Employing a novel assay to assess the presence of GPC-1 in UCS by use of MIL-38 staining and detection by IFA, prostate cancer patients could be distinguished from normal controls and patients with BPH. The assay showed high specificity in discriminating between prostate cancer, BPH, and normal control patients, and performed particularly well in this regard for patients with PSA values ≥4ng/ml. These data demonstrate the potential of GPC-1 as a biomarker for prostate cancer.

The current study aimed to examine the feasibility of an IFA assay employing a monoclonal antibody for specific detection of a biomarker in UCS, for the identification of prostate cancer. The assay successfully detected the biomarker GPC-1 in UCS, however, the study highlighted significant challenges associated with the analysis of biomarkers in urine cell preparations (reviewed in depth in [14]). For example, the variability in amount of cellular material collected from the urine poses a challenge. The prostate cancer cellular content of the urine may be improved by the use of DRE prior to urine collection. Indeed, DRE is currently used prior to PCA3 testing to improve the performance of the test, increasing the percentage of valid results obtained from 80% without DRE to 98% with the procedure [15]. However, although DRE may address low prostate cancer cell numbers in the urine sediment, variability in total cell number persists as a challenge using this method [8]. The use of UCS analysis for the diagnosis of prostate cancer poses challenges that must be addressed to enable its future clinical use.

While technically challenging, the detection of GPC-1 using the MIL-38 IFA assay described here shows promise for improving detection of prostate cancer beyond the current standard PSA. The assay distinguished between prostate cancer and BPH with a sensitivity of 71% and a specificity of 76%. Importantly, the specificity of the assay increased as PSA levels increased, with the highest specificity observed for patients with PSA ≥4ng/ml. This may improve on the current PSA test, for which specificity is low where PSA is high (32%) [3]. In addition, the sensitivity of the assay increased as serum PSA levels decreased, with the highest sensitivity reported for patients with serum PSA levels <2 ng/ml. Importantly, the MIL-38 IFA had a relatively high sensitivity and specificity in the clinically “grey zone” of PSA values (4-10ng/mL) as compared to PSA alone. The overall specificity of the use of the combined markers was better than their use individually. The Prostate Health Index (PHI) has low specificity (31.1%) where sensitivity is high. [16]. Thus, the combination of the MIL-38 assay with PHI may improve specificity.

The results described herein demonstrate the potential of GPC-1 as a biomarker for prostate cancer. GPC-1 detection in UCS was a particularly good predictor in patients with PSA values below 4 ng/ml. The combined use of GPC-1 with other known urine biomarkers for prostate cancer may improve the detection of prostate cancer beyond the use of PSA alone. The detection of GPC-1 in urine or blood, perhaps in combination with PSA and/or other biomarkers, may provide clinical benefit in terms of less invasive testing, earlier and more accurate detection of prostate cancer, and decrease the number of unnecessary biopsies. The potential utility of the IFA test presented herein, following further development, should be tested in larger cohorts of patients, and compared to currently available tests such as PCA3 and PHI for sensitivity and specificity. Moreover, it would be of interest to look at the effect of potential combinations of GPC-1 expression with other biomarkers, such as body mass index and prostate volume, on test performance. Clearly, detection of GPC-1 using MIL-38 has potential as a diagnostic approach for prostate cancer. We are currently investigating detection of the soluble form of GPC-1 in blood for this reason.

There is a significant clinical need for a means of discriminating between aggressive and non-aggressive prostate cancer in patients presenting with high PSA, particularly in the 2-10ng/ml range. There is currently an unacceptable risk of overdiagnosis leading to unnecessary treatment of non-aggressive cancers. High expression levels of GPC-1 have been associated with an aggressive phenotype in some malignancies, for example, high tumour expression of GPC-1 was associated with poor prognosis in patients with oesophageal squamous cell carcinoma (ESCC) and in another study, pancreatic ductal adenocarcinoma (PDAC) [17, 18]. Measurement of GPC-1 expression levels, rather than GPC-1 positivity *per se*, may be a means of distinguishing between aggressive and non-aggressive cancer. This could be assessed using a modified IFA assay able to quantify GPC-1 fluorescent signal, and normalising to prostate cancer cell content using PSA expression (as is used for normalization in the PCA3 test [19]). This should be investigated if the IFA test were to be further developed. An alternative, and simpler, approach, would be quantification of soluble GPC-1 levels in blood. We have developed such a test (manuscripted accepted) and are currently investigating the ability of soluble GPC-1 in blood to distinguish aggressive from non-aggressive cancer.
